# Regional lung densities in alpha-1 antitrypsin deficiency compared to predicted values

**DOI:** 10.1186/s12931-019-1012-3

**Published:** 2019-02-28

**Authors:** Berend C. Stoel, Jan Stolk, M. Els Bakker, David G. Parr

**Affiliations:** 1 Division of Image Processing, Department of Radiology, Leiden University Medical Center, Leiden, The Netherlands; 2 Department of Pulmonology, Leiden University Medical Center, Leiden, The Netherlands; 3 Department of Respiratory Medicine, University Hospitals of Coventry and Warwickshire, Clifford Bridge Road, Coventry, UK

**Keywords:** Computed tomography, Emphysema, Image analysis, Outcome measures, Standardization

## Abstract

**Background:**

We developed a method to calculate a standard score for lung tissue mass derived from CT scan images from a control group without respiratory disease. We applied the method to images from subjects with emphysema associated with alpha-1 antitrypsin deficiency (AATD) and used it to study regional patterns of differential tissue mass.

**Methods:**

We explored different covariates in 76 controls. Standardization was applied to facilitate comparability between different CT scanners and a standard Z-score (Standard Mass Score, SMS) was developed, representing lung tissue loss compared to normal lung mass. This normative data was defined for the entire lungs and for delineated apical, central and basal regions. The agreement with D_LCO_%pred was explored in a data set of 180 patients with emphysema who participated in a trial of alpha-1-antitrypsin augmentation treatment (RAPID).

**Results:**

Large differences between emphysematous and normal tissue of more than 10 standard deviations were found. There was reasonable agreement between SMS and D_LCO_%pred for the global densitometry (κ = 0.252, *p* < 0.001), varying from κ = 0.138 to κ = 0.219 and 0.264 (*p* < 0.001), in the apical, central and basal region, respectively. SMS and D_LCO_%pred correlated consistently across apical, central and basal regions. The SMS distribution over the different lung regions showed a distinct pattern suggesting that emphysema due to severe AATD develops from basal to central and ultimately apical regions.

**Conclusions:**

Standardization and normalization of lung densitometry is feasible and the adoption of the developed principles helps to characterize the distribution of emphysema, required for clinical decision making.

**Electronic supplementary material:**

The online version of this article (10.1186/s12931-019-1012-3) contains supplementary material, which is available to authorized users.

## Background

Chronic obstructive pulmonary disease (COPD) is defined physiologically using spirometric measurement of forced expiratory volume in 1 s (FEV_1_), forced vital capacity (FVC) and the ratio of FEV_1_/FVC [1]. Pulmonary emphysema is frequently present in patients with COPD and may be assessed by measuring the diffusing capacity for carbon monoxide (D_LCO_), which reflects the emphysematous tissue destruction that leads to loss of alveolar structure and, as specifically reflected in the D_LCO,_ the pulmonary vascular bed [2]. The time course of D_LCO_ and FEV_1_ decline as physiologic parameters of emphysema progression is highly variable between (and within) patients and they correlate poorly [3]. Although D_LCO_ is considered to reflect emphysema severity in patients with COPD, emphysema is defined in histopathological rather than physiological terms [4] and a more disease-specific parameter, obtained from lung densitometry using computed tomography (CT), was introduced 40 years ago [5, 6], and validated against histopathological standards by three different laboratories [7–9]. Lung densitometry was also validated by relating densitometry to clinically relevant measures [10–13]. It was found to be more consistent over time as compared to FEV_1_ and D_LCO_ [14], most probably because densitometry is a more direct measurement of emphysema and intrinsically effort independent. In patients with emphysema associated with alpha-1-antitrypsin deficiency (AATD), D_LCO_/VA predicted all cause and respiratory mortality. However, CT densitometry consistently proved to be the best independent predictor of mortality [15]. Some years later, the European Medicines Agency (EMA) approved phase II and III randomized controlled clinical trials to study the effect of new drug treatments on emphysema and, in 2007, the United States Food and Drug Administration (FDA) accepted the methodology for use as an outcome measure in trials of disease modifying therapy in AATD patients. In 2015, the EMA approved a license for Respreeza on the basis of a beneficial treatment effect demonstrated using lung CT densitometry [16–18]. As post-hoc analysis, regional densitometry has been introduced to study emphysema progression and treatment effects in the apical, central and basal regions of the lungs [19–21] to improve insight into pathophysiology and local emphysema treatment planning.

The clinical application of lung densitometry, however, has not followed the pace of its application in clinical research. To date, there is no international accepted database with reference values obtained from individuals with healthy lungs and no standardized CT image acquisition protocol for lung densitometry. Moreover, we currently lack adequate standardization between different CT manufacturers (despite calibration for water and air), correction of lung density for differences in lung sizes between subjects and for inspiration levels [22].

The aim of our study was to develop an integrated method to report lung density in terms that would address the above obstacles and facilitate the introduction of the methodology into routine clinical practice [23].

## Methods

### Overview

**Fig. 1:**
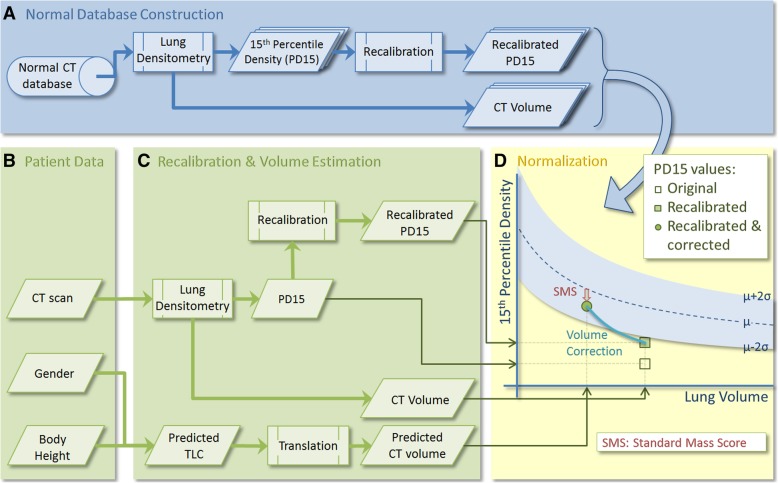
Overview of the proposed integrated method. **a** Construction of the normal database, producing lung volumes and recalibrated density values from a normal population; **b** Input data from an emphysema patient to be evaluated; **c** Calculation of recalibrated density data and predicted CT lung volume of a patient; **d** Normalization and volume correction of the density values, resulting in a Z-score for lung tissue mass: Standard Mass Score (SMS). In this particular example, a seemingly abnormally low lung density (PD15) can actually fall within the normal range for tissue mass, after accounting for different CT scanners (i.e. recalibration of lung density values) and lung volumes (volume correction)

We considered that to express CT lung density as “percent predicted density” values would require: 1) a recalibration method for compensating for differences between CT scanners; 2) a comparison with normal values from a database, producing a standard score; and 3) a method to correct for volume differences within and between subjects (see Fig. 1).

First, all CT data from normal controls were analyzed by lung densitometry (Fig. 1a), producing for each subject the lung volume during CT scanning and a recalibrated density value, 15th percentile density (PD15). From this database, the mean relation between lung size (volume) and PD15 in the normal population was determined, along with the standard deviation in density at each volume.

A standard score for a particular patient was produced from CT imaging, together with gender and body height (Fig. 1b). This CT scan was analyzed by densitometry software, to produce a lung CT volume and a recalibrated PD15 value (Fig. 1c). In the same step, the predicted total lung capacity (TLC) was calculated (based on gender and body height) and translated into a predicted lung CT volume, which is needed because of the difference in definition between ‘physiologically’ measured TLC and ‘anatomically’ measured CT-volume, the latter of which is defined as the total number of voxels in the lungs, excluding the trachea but including parenchyma and small vessels. In the final step (Fig. 1d), a so-called standard mass score (SMS) was calculated, based on the normal density database and the patient’s lung volume and recalibrated density values. Volume correction was applied based on the predicted CT volume.

In addition to these global measurements, the same approach was followed for regional lung densitometry, where the apical, central and basal regions of the lungs were analyzed separately [19]. To define these areas, the lungs were divided vertically into 12 equivolumetric partitions and the superior and inferior partition were omitted, as they contain artifacts, e.g. partial volume effects [20]. The separate regions were determined by combining the remaining partitions (apical:3, central:3, basal:4). The above normalization was also applied to these separate regions in the normal database, producing three regional standard mass scores; i.e. SMS_apical_, SMS_central_, SMS_basal_.

A detailed description of the different components is presented in the following sections.

### Lung densitometry

All CT data were analyzed by a software package Pulmo, version 2.1 (Medis specials, Leiden, the Netherlands), using a threshold of − 380 HU to detect both lungs, with exclusion of the trachea [22]. From this segmentation result, the density distribution was calculated and lung density was measured as the 15th percentile density^1^ (PD15; the threshold density in gram/liter, at which 15% of lung voxels have a lower density). By summing all included lung voxels, the lung volume (CT-volume) was determined.

### Normal CT database

CT scans from 76 subjects (52 males, 24 females, see Table 1) without respiratory symptoms, who had been screened for pulmonary metastases following treatment of osteosarcoma, were reviewed by a radiologist and, if a normal radiological appearance was observed, the subjects were checked for normal spirometry and gas transfer, as reported in an earlier study [24]. In this population, FEV_1_ (standard deviation, SD) and D_LCO_(SD) was 93(4) % and 95(10) % of predicted values, respectively. Of these subjects, 16 were current smokers, 26 were ex-smokers and 34 were never-smokers. Age ranged between 26 and 78 years. The CT data were acquired using a Philips AVE-U scanner (Phillips Medical Systems, Eindhoven, the Netherlands) at full inspiration and were used to create a database of normal values (normal database). The CT scanner was calibrated as recommended by the manufacturer using a standardized image acquisition protocol (at 140 kVp, 40 mAs, pitch factor 2, 7 mm collimation, reconstructed with a slice thickness of 7 mm, 5 mm increment and reconstruction filter 4 [24]). CT images were analyzed, and both lung volume and PD15 values were stored in a database.

**Table 1:** Characteristics of the normal subjects [24] and AATD patients [16]

	Normals	AATD
Subjects [n]	76	180
Age [years]	44.5(11.8)	53.1(7.4)
Sex [M/F]	52/24	98/82
BMI [kg/m^2^]	25(3)	26(4)
FEV_1_ [%pred]	93(4)	47(12)
FEV_1_/VC	0.87(0.08)	0.44(0.11)
D_LCO_[%pred]	95(10)	55(19)

To identify those variables required for normalization, the influences of several parameters on percentile density were explored in this normal database, by linear regression using log-transformed percentile density as response variable. The potential explanatory variables included gender, smoking status, age, log-transformed CT-volume and inspiration level, defined as the log ratio of CT-volume and predicted total lung capacity (TLC). Data were analyzed with SPSS 16.0 (SPSS Inc., Chicago, IL) and a stepwise variable selection was applied. Age, smoking status and gender were excluded from the model, as they did not significantly explain additional variation. The model with only CT-volume and inspiration level fitted best to the data (R^2^ = 0.65).

Therefore, lung size and inspiration level were used for normalization of PD15 values from patients, performed in three steps, as discussed in the following three sections: 1) recalibration of density values; 2) correction for inspiration level; and 3) comparison with the normal database with lung size as covariate.

### Recalibration

Percentile density values were recalibrated to account for differences that occur between different CT scanner types and manufacturers despite routine calibration for air and water. In an internal recalibration method, all density values were rescaled during image analysis, using the mean air density sampled outside of the patient (above the sternum) as a reference value for air [25]. The mean density measured in the descending aorta was used as a reference for the density of blood (rescaled to 1050 g/L) [22].

### Volume correction

Correction for variation in inspiratory level has been explored using several methods and adapted for use in drug evaluation trials [11]. For analyzing data from single time points, only a physiological method can be used, referred to as the ‘sponge model’. In this model, differences in inspiratory level are considered to be mass-preserving, i.e. lung mass remains constant during the respiratory cycle, as in a dry sponge that is compressed then released [26, 27]. As a result, lung volume and density are linearly related when both are log transformed, with a slope of exactly − 1. Consequently, this linear relation is used to correct for differences in inspiratory level, by calculating the percentile density that would apply if the patient had inhaled to his/her predicted total lung capacity (TLC_pred_). In contrast to pulmonary function tests, however, patients are scanned in the supine position and, by definition, CT-volume includes lung tissue and excludes tracheal air, whereas TLC is a measure of total air volume without lung tissue, but including tracheal air. Therefore, the predicted CT-volume (V_CT,pred_) was estimated from the predicted TLC values from the normal population by linear regression, separating for gender. The resulting coefficients for intercept and slope, γ and δ, respectively, were then used to translate between predicted TLC and predicted CT-volumes.

Because of image reconstruction errors, possible physiological influences and the fact that the PD15 is used instead of the mean density values, the ‘sponge model’ does not apply exactly in practice [28], therefore a steeper slope (S) of − 1.1 was used, obtained from optimizing the reproducibility of the volume correction, using the baseline inspiratory and expiratory scans of the RAPID trial.

In short, the corrected percentile density value, ρ_cor_, was defined as:

1 $$ {\rho}_{cor}={\rho}_{cal}\bullet {\left(\frac{V_{CT, pred}}{V_{CT}}\right)}^S={\rho}_{cal}\bullet {\left(\frac{\gamma +\delta \bullet {TLC}_{pred}}{V_{CT}}\right)}^S $$

where ρ_cal_ is the recalibrated percentile density value, V_CT_ and V_CT,pred_ are the observed and predicted lung volume in CT, respectively, and TLC_pred_ the percent predicted value according to the ERS standard, based on body height and gender [29]. The coefficients γ and δ are the intercept and slope from linear regression, respectively, to translate TLC predicted values to normal CT volumes.

### Normalization

For the final step, the recalibrated and volume-corrected percentile density was compared to the database of normal values from an earlier study [24], the raw data of which is made available in the Additional file 1. To account for differences in lung size, data were corrected based on the linear relation between log-transformed volume and log-transformed density. To indicate the ‘percent predicted density’ for a particular patient, the standard score (Z-score) was calculated, defined by the difference between the measured percentile density and the predicted value (derived from the normal database) at the patient’s lung CT volume, divided by the residual standard deviation after linear regression.

Pulmonary emphysema is characterized by a reduction in lung mass (due to tissue loss and reduced blood volume in pulmonary capillaries). The use of volume-corrected data reflects the loss of tissue mass alone compared to the normal database and may be referred to as the ‘Standard Mass Score’ (SMS).

An SMS of 0 is equivalent to normal tissue mass (“100% predicted”). A value between − 2 and 0 indicates a decreased lung tissue mass that is still within the normal range, and a value between 0 and 2 indicates a normal but increased tissue mass. All SMS values above 2 or below − 2 indicate an abnormal increased or decreased lung tissue mass, respectively.

Thus, the recalibrated and volume-corrected standard mass score was defined as:

2 $$ SMS=\frac{\log \left({\rho}_{cor}\right)-\left(\alpha +\beta \kern0.28em \log \left({V}_{CT, pred}\right)\right)}{\sigma }=\frac{\log \left({\rho}_{cor}\right)-\left(\alpha +\beta \kern0.28em \log \left(\gamma +\delta \kern0.28em {TLC}_{pred}\right)\right)}{\sigma }, $$

where log(ρ_cor_) and α + β· log(V_CT,pred_) are the measured and predicted log percentile density values, respectively, α and β are the intercept and slope from the regression line between log volume and log percentile density from the CT scans of normal subjects, and σ is the residual standard deviation after linear regression.

### AATD CT database

Baseline CT scans were selected from the RAPID clinical trial database (see Table 1), described previously [16, 17]. In this study, patients aged 18–65 years were included with serum AAT levels below 11 μM and FEV_1_ -values ≥35% and ≤ 70% of predicted. Participants were excluded if they had smoked tobacco within 6 months prior to the start of the study, had undergone lung transplantation, lobectomy or lung volume reduction therapy, or had selective IgA deficiency.

CT scanning was performed using a standardized CT acquisition protocol, optimized for lung densitometry, at total lung capacity (TLC) and functional residual capacity (FRC), where only the TLC scan was used for the current study [17].

### Validation

To validate the method in terms of (regional) structure-function relationship [30], the Spearman correlation was studied between standard mass score and D_LCO_ percent predicted values, and FEV_1_ percent predicted from the AATD patient group, respectively. The agreement in separation into normal and abnormal lung structure or gas exchange was assessed by kappa-statistics. In this separation, SMS values less than − 2 were considered abnormal, and for D_LCO_ a threshold of 80% of predicted values was used to define the lower limit of the normal range. A significance level of 0.05 was used for all statistical tests.

As an internal validation using the control data, a leave-one-out cross-validation experiment was performed, where the normalization was determined with n-1 controls and tested on one, with n-1 different combinations.

The distribution of SMS values across the basal, central and apical regions was used to distinguish between different subgroups in the RAPID study population. Subsequently the differences in D_LCO_%pred between these subgroups was tested.

## Results

**Fig. 2:**
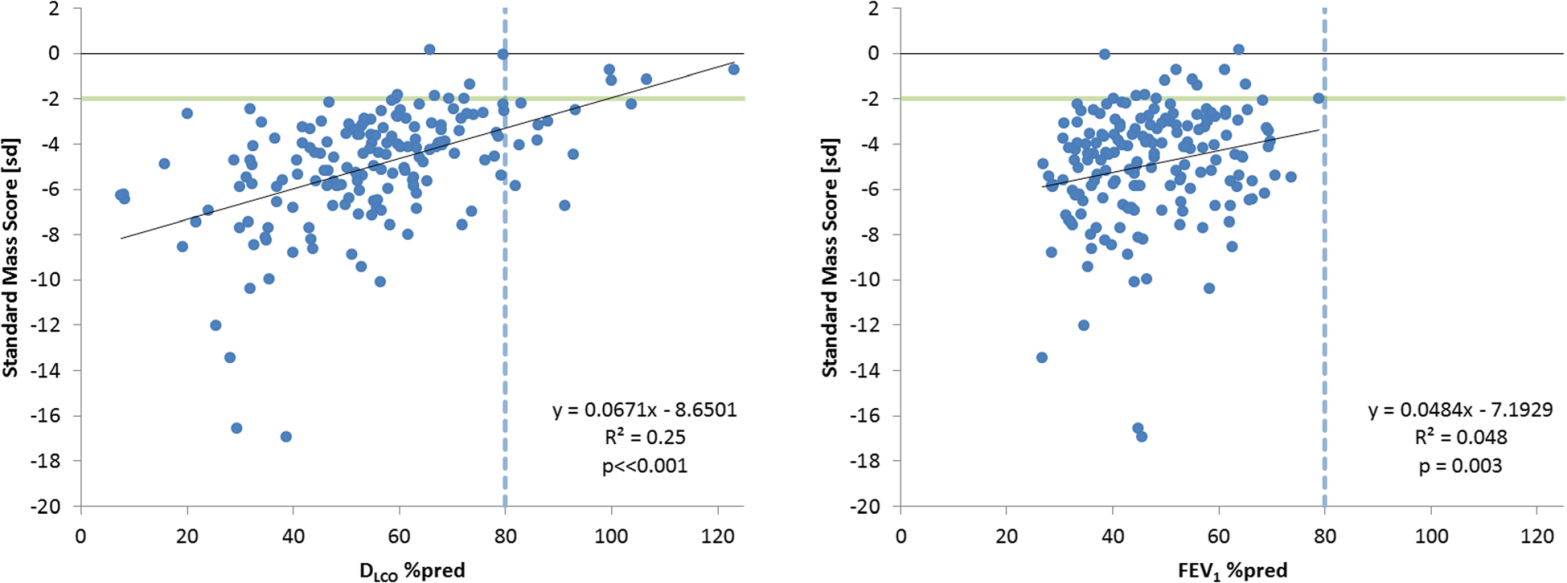
Relation between standard mass score and percent predicted values for D_LCO_ and FEV_1_. The green horizontal line indicates the lower limit of normal SMS; the dotted vertical line indicate lower limit of normal pulmonary function. Note that no normal FEV_1_ values (> 80%) were observed because 70% predicted was an exclusion criterion for the RAPID trial (natural variation in FEV_1_ values causes the occurrence of baseline values above the exclusion criterion)

**Fig. 3:**
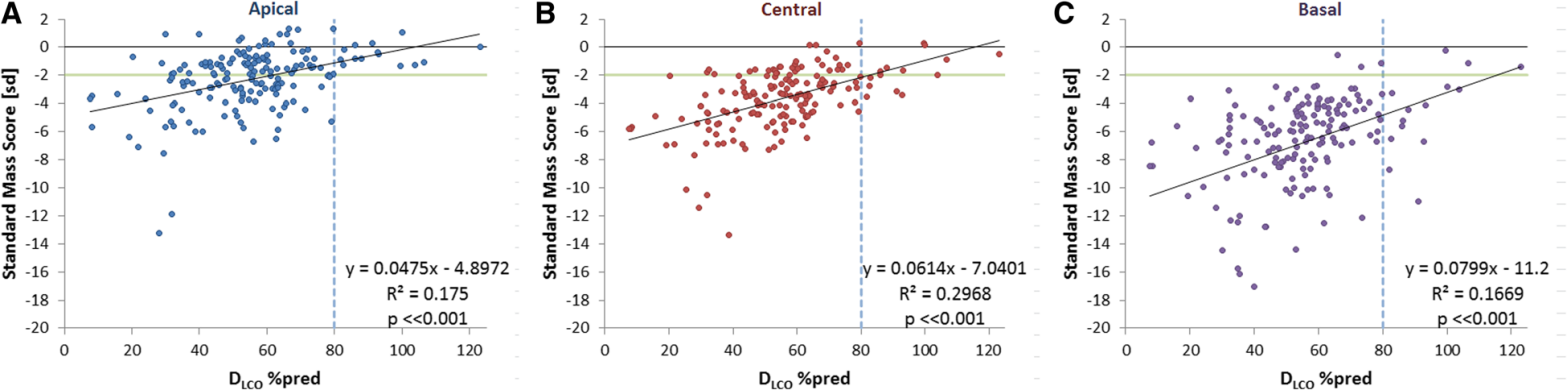
Correlations between SMS and D_LCO_%pred in: **a**. Apical; **b**. Central; and **c**. Basal region. The green horizontal line indicates the lower limit of normal SMS; the dotted vertical line indicate lower limit of normal pulmonary function

The correlations of SMS with the %-predicted values for D_LCO_ and FEV_1_ are presented in Fig. 2. The correlation with D_LCO_ was moderate, but statistically significant (R^2^ = 0.25, *p* < < 0.001); and for FEV_1_ the correlation was weak, R^2^ = 0.048 (*p* = 0.003**)**. For comparison, the correlation between the %-predicted values for D_LCO_ and FEV_1_ was statistically significant but also weak, R^2^ = 0.1202, *p* < < 0.001. For the apical, central and basal regions the correlations between D_LCO_ %pred and SMS values are shown in Fig. 3. It was noted that there were three patients with exceptionally low D_LCO_ %pred values below 10%, which we considered to be likely outliers. Since omitting these values did not considerably change the correlation between SMS and D_LCO_ %pred (R^2^ = 0.26, *p* < < 0.001), it was decided to preserve these data.

The agreement between global SMS and D_LCO_ %pred in distinguishing abnormal structure or function is presented as a confusion matrix in Table 2. The kappa-statistic showed a fair agreement between SMS and D_LCO_%pred (κ = 0.252, *p* < 0.001), varying from κ = 0.138 to κ = 0.219 and 0.264 (p < 0.001), in the apical, central and basal region, respectively.

**Table 2:** Confusion matrix, SMS versus D_CLO_ %pred

		D_LCO_ %pred		
		normal	abnormal	Total
Standard Mass Score	normal	4	10	14
	abnormal	8	151	159
κ = 0.252, *p* < 0.001	Total	12	161	173

From the cross-validation with normal controls, we found that the average SMS value of unseen controls was − 0.05, with a standard deviation of 1.03, closely corresponded to the targeted mean and standard deviation of 0 and 1, respectively.

**Fig. 4:**
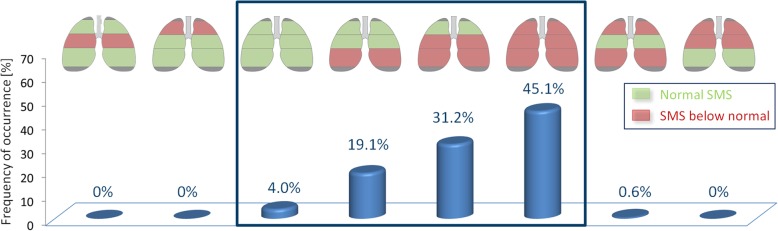
Bar plot of the frequency of occurrences of all possible combinations of normal and below-normal tissue mass, over the three different regions. Only a subgroup of four possible combinations predominantly occur in practice, as highlighted by the rectangular overlay

Analyzing the frequency of occurrence of the SMS values below normal across the different lung regions (apical, central, basal) in the AATD population revealed that from the eight possible pattern combinations only four predominantly occur, as shown in Fig. 4. In this figure, green regions represent normal lung mass, and red regions indicate SMS values below normal. In 4% of cases, the lungs actually had normal tissue mass, where the frequency of occurrence increases as the basal, central and apical regions become incrementally involved.

**Fig. 5:**
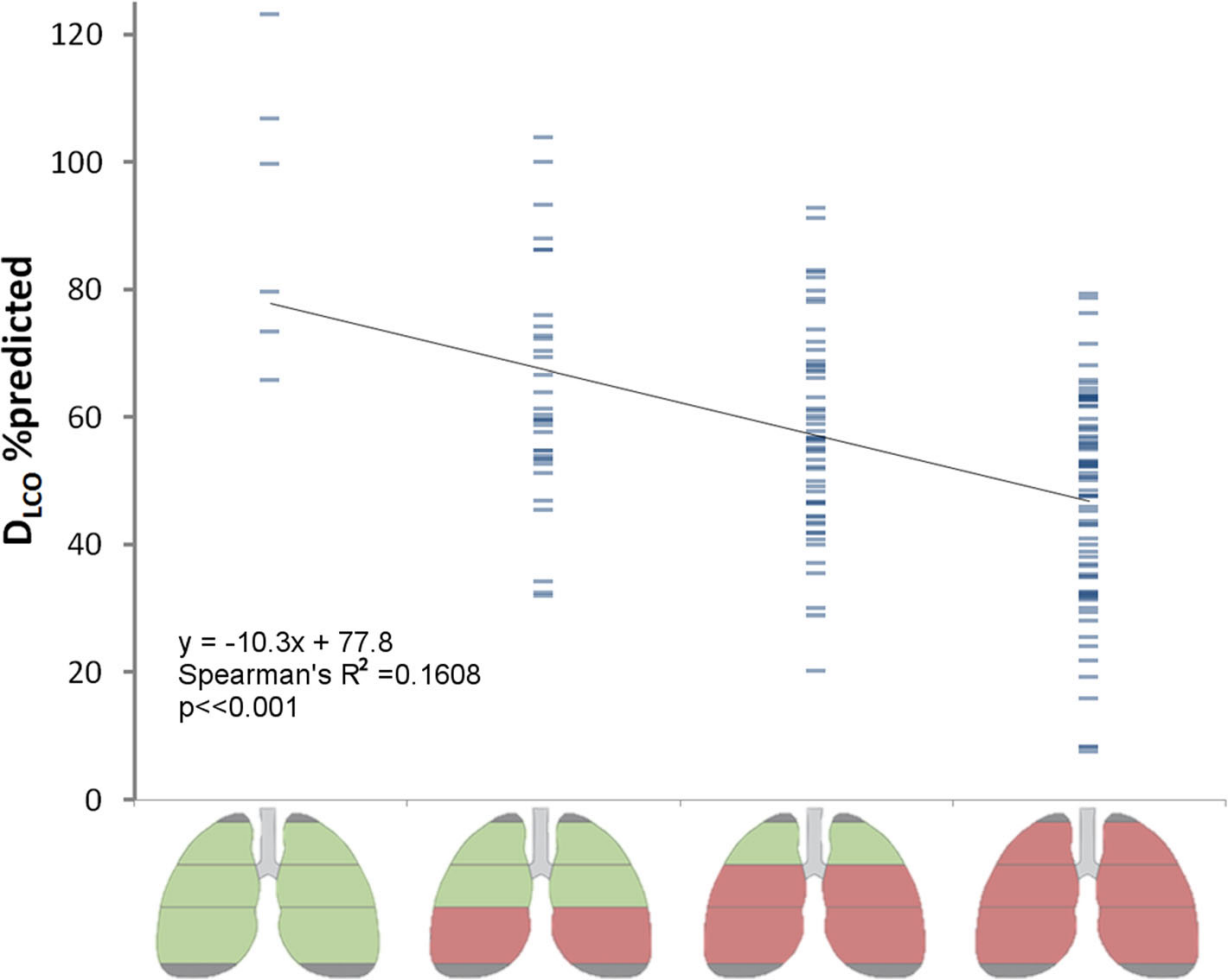
Distribution of D_CLO_ %pred values across the different SMS subgroups

The association between subgroups and D_LCO_%pred is shown in Fig. 5 (R^2^ = 0.16, *p* < < 0.001). The range in D_LCO_%pred was more comparable between the different subgroups in Fig. 5, than for the global SMS scores from Fig. 2, where the range in D_LCO_%pred was greater in the higher SMS scores.

## Discussion

The accepted principles of standard procedures for the measurement of pulmonary function also apply to lung densitometry, and require the use of the same equipment, preferably the same technician, the same protocol, an appropriate calibration, correction for uncontrolled covariates and a suitable reference to normal values. Most of these issues have been addressed in the research efforts of the past decades [26, 27, 31, 32] and, consequently, CT densitometry has been accepted as the primary outcome parameter in trials evaluating new drugs for pulmonary emphysema [33]. However, the variability between CT scanners and a lack of normative data has hampered its application in clinical practice [23]. In the present study, we have established an initial step in this process, by proposing a method to standardize between CT scanners, in combination with a correction for volume differences and a subsequent comparison with a normal database.

Demonstration of the large differences in standard mass score between emphysema patients and normal controls of more than 10 SD (see Fig. 2), and the stronger correlation between SMS and D_LCO_, than between D_LCO_ and FEV_1_, supports the application of CT lung densitometry in clinical practice. The future acquisition of a more extensive normal database would provide even greater confidence and support for the use of CT lung densitometry in routine clinical practice.

From the analysis of the normal database, we found that percentile density was not influenced by gender and age, which supports the data of Gevenois et al. [34], who studied the influence of different factors on the mean lung density in 42 healthy subjects. Therefore, any differences in lung density between genders should be interpreted with caution, since the difference is more likely to be a direct consequence of the gender-associated differences in lung size. Gevenois and colleagues [34] did find a slight influence of age on the relative area of low attenuation or ‘voxel index’ (which is an alternative measure to the percentile density method). However, this influence may also be explained by differences in lung size.

The first study in which normative density data were presented was by Kalender et al. [35], where spirometrically controlled CT data from 52 healthy subjects was analyzed. The mean lung density was calculated from three single slices and used as reference data. They concluded that spirometric control proved difficult even in cooperative patients, preventing an objective comparison with normal controls. Marsh et al. [36] compared CT densitometry from 22 emphysema patients with a normal database of 185 normal subjects, and found a poor discriminating ability of the relative area. However, both studies employed single slice protocols so that the lung volume during scanning could not be measured and a posteriori volume correction was therefore not possible. Heussel et al. [37] compared patients with COPD and interstitial lung disease, employing 44 patients with COPD GOLD stage 0 used as normal data, and concluded that a 15th percentile density higher than − 950 HU should be considered normal. More recently, Mets et al. [38] and Pompe et al. [39] presented normal ranges for the 15th percentile density from a cohort of 70 young male divers with above-normal spirometry and of 250 current or former male smokers with normal gas transfer, respectively. In the above studies, no volume correction or standardization was applied, even though volumetric data was available, thereby hampering interpretation and comparison with our data.

Some limitations apply to the current study, and refinements of the methods may still be needed. The recalibration method may be too simple to reflect all potential differences between scanners, such as difference in image reconstruction, different degrees in beam hardening effects and subsequent correction. Alternative methods for performing recalibration have been proposed in the literature, including the use of dedicated lung phantoms [40] or by the normalization of the entire appearance of the CT scans based on frequency band decomposition [41]. This latter method requires, however, that different reconstructions from the same patient are available to obtain the normalization function, which limits its practical implementation. Further standardization of CT image reconstruction is still required to make lung densitometry even more accurate. Therefore, the initiative of the scientific advisory board of the Radiological Society of North America (RSNA) to establish CT standardization, as part of the “Quantitative Imaging Biomarker Alliance” (QIBA) is essential for further acceptance of CT densitometry as a clinical tool [42].

The size of the normal database used here is limited because, for ethical reasons, we were only able to scan patients in this cohort if they were considered at risk for pulmonary metastases. Therefore, the collection of normal values took a long time period and, consequently, the scanner used to develop this data is no longer ‘state-of-the-art’. Notwithstanding this potential limitation, there is no indication that older CT scanners produce less accurate densitometric results than modern scanners. Further multi-center studies are needed to extend this database and produce more relevant standard scores for emphysema, possibly involving more explanatory variables in the statistical model. For example, smoking status is a known factor influencing lung density in a group of 463 COPD patients [43], and may need to be included in the model.

Nevertheless, the requirements for the highest degree of precision are less important for cross-sectional studies compared to longitudinal studies of potential disease modifying treatment effects. However, simple application and refinement of the current methodology will facilitate such studies.

The presented method enabled also a regional analysis of lung tissue mass compared to normal tissue, in a representative sample of patients with severe AATD. The finding that only four subgroups exist based on their regional SMS patterns suggests that emphysema in AATD develops from basal to global destruction of lung tissue, which needs to be confirmed in longitudinal studies.

## Conclusions

The standardization and normalization of lung mass values has been shown to be feasible. Consequently, the methodology could be used in clinical practice in the near future, although further refinement of the standardization methods may be needed, either by a posteriori recalibration or by a standardized protocol for different CT manufacturers. The adoption of these principles may improve the application of lung CT densitometry as a research and clinical tool where information about distribution of emphysema is required for the purpose of clinical decision making.

## Additional file

Additional file 1: Individual data from the normal database. (XLSX 13 kb)

## Abbreviations

AATD: Alpha-1 antitrypsin deficiency
COPD: Chronic obstructive pulmonary disease
CT: Computed tomography
DLCO: Diffusing capacity for carbon monoxide
EMA: European medicines agency
FDA: United states food and drug administration
FEV1: Forced expiratory volume in 1 s
FVC: Forced vital capacity
MLD: Mean Lung Density
PD15: 15th Percentile density
QIBA: Quantitative imaging biomarker alliance
RSNA: Radiological society of North America
SMS: Standard Mass Score
TLC: Total lung capacity
VA: Alveolar volume

## Footnotes

1 The 15th percentile density was chosen over mean lung density (MLD), since densities are not normally distributed and MLD is more prone to outliers from the segmentation, affecting its reproducibility [44, 45].
